# Clinical analysis of 82 cases of acute promyelocytic leukemia with *PML-RARα* short isoform in children and adults

**DOI:** 10.3389/fonc.2024.1342671

**Published:** 2024-02-21

**Authors:** Qiaolin Huang, Yicheng Zhang, Miao Zheng

**Affiliations:** ^1^ Department of Hematology, Tongji Hospital, Tongji Medical College, Huazhong University of Science and Technology, Wuhan, Hubei, China; ^2^ Immunotherapy Research Center for Hematologic Diseases of Hubei Province, Wuhan, Hubei, China; ^3^ Key Laboratory of Organ Transplantation, Ministry of Education, National Health Commission (NHC) Key Laboratory of Organ Transplantation, Key Laboratory of Organ Transplantation, Chinese Academy of Medical Sciences, Wuhan, Hubei, China

**Keywords:** acute promyelocytic leukemia, *PML-RARα* short isoform, bcr3 isoform, clinical features, gene mutation

## Abstract

**Background:**

Acute promyelocytic leukemia (APL) with *PML/RARα* fusion gene is a distinct variant of acute myeloid leukemia. According to the different break sites of the *PML* gene, there are three transcripts: Long (bcr1), Variant (bcr2) and Short (bcr3).

**Methods:**

We retrospectively analyzed 82 APL cases with *PML-RARα* short isoform.

**Results:**

A total of 384 patients with APL were seen, of which 85(22.14%) had *PML/RARα* short isoform (bcr3) and 82 met the inclusion criteria. The median age was 33.5 years (range, 2-72 years). The incidences of hemorrhage in the intermediate- and high-risk group were higher, but only the incidence between medium and low risk differed statistically (*P=0.006)*, and the incidences of fever, fatigue, splenomegaly, and lymph node enlargement and differentiation syndrome (DS*)* in those groups were not statistically significant (*P*>0.05). *FLT3* gene mutation rate and the mortality rate of the high-risk group were significantly higher than that of other groups (*P*=0.040 and *P*=0.004, *P=*0.041 and *P*=0.037, respectively). The mortality rate was lowest (4.26%) in the group treated with ATRA combined with arsenic and anthracycline. The 3-year OS and the 3-year DFS of the low and intermediate-risk group were better (*P*=0.019 and *P*=0.017, respectively).

**Conclusions:**

ATRA combined with arsenic and anthracycline had significant impact on outcomes in APL with *PML-RARα* short isoform.

## 1 Introduction

AML possesses a special type called acute promyelocytic leukemia (APL), accounting for 10-15% of the newly diagnosed acute myeloid leukemia (AML) cases, which is characterized by anemia, infection, bleeding tendency and other symptoms, and is prone to be complicated with abnormal coagulation function and extensive systemic hemorrhage (1). In the majority of APL patients, *PML/RARα* fusion gene is positive, commonly t(15; 17)(q22; q12-21), which is formed by the translocation and fusion of promyelocytic leukemia (*PML*) gene (located on chromosome 15, q22) and retinoic acid receptor alpha (*RARα*) gene (located on chromosome 17, q12-21). In addition, the PML gene has different break sites and fuses with the *RARα* gene to form three APL subtypes, long (bcr1), variant (bcr2) and short (bcr3), respectively (2).

*FLT3* serves as a transmembrane tyrosine kinase receptor involved in cell proliferation. Many APL patients, about 30%-40%, have *FLT3* mutation, which is often associated with higher WBC counts and bcr-3 breakpoints of *PML* gene, but has no effect on survival (3). The key to induction treatment is all-trans retinoic acid (ATRA) combined with arsenic, which helps most patients (90%) achieve complete remission (CR) (4). However, some APL patients experiencing induction therapy tragically died of severe intracranial hemorrhage, thrombotic events or infection, especially for APL patients with bcr3 isoform (5).

Studies about bcr3 isoform are limited, while there are many studies concentrated on *PML-RARα* transcript bcr1 isoform. Therefore, our cases mainly focus on the bcr3 isoform that is more inclined to bleeding, higher risk stratification, and poorer clinical prognosis (5). This study evaluated the response of APL patients with *PML-RARα* short isoform from different risk stratification to early induction therapy. We identified 82 APL patients with *PML-RARα* short isoform in our center from May 2012 to September 2023, and analyzed the clinical characteristics, response to early treatments and outcomes.

## 2 Materials and methods

### 2.1 Patients

82 APL patients with bcr3 isoform who were commited to Tongji Hospital from May 2012 to September 2023 were collected. The inclusion criteria included: (1) Patients with newly diagnosed acute promyelocytic leukemia meeting the diagnostic criteria of APL in the *Guideline for Diagnosis and Treatment of Hematologic Diseases* (6). (2) Patients with clear genotyping of *PML-RARα* short isoform. The exclusion criteria included: (1) Patients with secondary APL; (2) Patients who were not first treated in our hospital; (3) Patients who died before treatment; (4) Patients with other hematologic diseases or other systemic tumors.

We gathered all detailed information including clinical manifestations, blood routine and biochemical tests, flow immunophenotype analysis, chromosome karyotype analysis, bone marrow biopsy, *PML-RARα* gene detection, time of admission and discharge, outpatient treatment and clinical data of the whole process of diagnosis and treatment. The patients were divided into 3 risk groups according to the Sanz risk groups originating from the peripheral blood leukocyte and platelet counts at the initial diagnosis of APL: low-risk group (WBC<10×10^9^/L, PLT ≥40×10^9^/L), intermediate-risk group (WBC<10×10^9^/L, PLT40<10^9^/L), high-risk group (WBC≥10×10^9^/L).There were 15 patients in the low-risk group, 35 in the intermediate-risk group, and 32 in the high-risk group.

This study was approved by the ethics committee of Tongji Medical College, Huazhong University of Science and Technology. Written informed consent was waived because of the retrospective nature of the study.

### 2.2 Treatment

Induction treatment followed the guidelines for the management of acute promyelocytic leukemia, those patients were given all-trans retinoic acid (ATRA) with or/and arsenic agents or/and anthracycline medications until remission, totaling approximately 1 month. In this study arsenic agents included intravenous arsenic trioxide (ATO) or oral compound realgarindigo naturalis formula (RIF) which is a kind of Chinese patent medicine containing arsenic. The anthracycline drugs used in our patients were pirarubicin, idarubicin (IDA), and daunorubicin (DNR). The patients’ drugs were adjusted according to their specific condition. The clinical efficacy of patients was evaluated according to the *AML* IWG response criteria (7). The consolidation and maintenance treatments and central nervous system(CNS) prevention and treatment schemes were referred to the “Guideline for the Diagnosis and Treatment of Hematologic Diseases” (6).

### 2.3 Observation indicators

(1)Comparison of clinical characteristics of patients in different Sanz risk groups
(2) Induction stage and recurrence of patients
(3) Comparison of early efficacy of different treatment schemes
(4)Comparison of survival in patients with different risk groups
(5)Comparison of disease-free survival in patients with different risk strata

### 2.4 Efficacy assessment and follow-up

Early death was defined as patients who died before reaching complete remission (CR) during the induction phase of treatment. Hematologic complete remission (HCR), molecular complete remission (MCR) referred to IWG criteria (8). Overall survival (OS) was defined as the time from the initial diagnosis of APL with PML-RARα short fusion gene to the patient’s death, and disease-free survival (DFS) was defined as the time from the patient’s achievement of HCR to the first relapse. The follow-up deadline was September 1, 2023 and progression of disease was used as the primary end-point of follow-up and the disease-free survival (DFS) was the secondary end.

### 2.5 Statistical analysis

SPSS25.0 statistical software was used to analyze the data. The numerical data were described as range, median and mean value. The enumeration data were expressed as [n(%)], and we used the Pearson chi-square test to deal with the enumeration data, or Fisher’s exact probability method was adopted if the expected frequency was<5. Life table methods and Kaptan-Meier survival curves were used for survival analysis, and the 3-year survival rate from initial diagnosis and 3-year disease-free survival from complete remission were counted, and comparison of survival among groups was performed by Log-rank test. All tests were 2-tailed and P<0.05 was considered statistically significant.

## 3 Results

### 3.1 Comparison of clinical characteristics of patients in different risk groups

We collected a total of 384 patients with APL, of which 85(22.14%) had *PML-RARα* short isoform but 82 met the inclusion criteria and were analyzed and 3 of the 85 patients identified with bcr3 met the exclusion criteria, 1 of whom died before starting treatment, and 2 of whom came to our hospital for treatment after a relapse, not for the first time. There were 42 males and 40 females, aged 2-72 years, with a mean age of 35 years and a median age of 33.5 years.

In patients newly diagnosed APL with bcr3 isoform, the main clinical features included bleeding, fever, fatigue, splenomegaly and lymph node enlargement. Bleeding (62.20%) was the most common presentation, followed by fever (45.12%). Fatigue manifestation was seen in 41.46% of patients. No differences in the incidence of fever, fatigue, splenomegaly and lymphadenopathy among three risk groups at initial diagnosis were found (*P*>0.05), while only the incidence of bleeding showed difference (*P*<0.05) among different risk groups. Comparing the incidence of bleeding among different risk groups, the intermediate-risk group and the high-risk group were higher than the low-risk group. There was a statistically significant difference in the bleeding incidence between the intermediate-risk group and the low-risk group (*P*=0.006), but no difference was found in other pairwise comparison groups(*P=0.062, P=0.299).*

During early induction therapy, 18 patients developed DS. The presentations of DS included breathing difficulties, weight gain etc. Of those 18 patients with DS, no one succumed to it. The incidences of DS among three risk groups were compared, but no differences were found among them (*P*>0.05) (**Table 1**). We demonstrated statistically that the difference in the occurrence of DS in the different drug treatment groups was not statistically significant(*P=0.556*). We compared the incidence of DS in the anthracycline-using and anthracycline-naïve groups and there was no statistical difference(*P=0.658*). We do not attribute the lower incidence of DS to the proportion of patients treated with anthracycline.

**Table 1:** Baseline patient characteristics.

characteristic	variable	Whole Group				Children
		low	Intermediate	high	P	
gender	male	9 (60.0%)	16 (45.7%)	17 (53.1%)	–	4 (50.0%)
	female	6 (40.0%)	19 (54.3%)	15 (46.9%)	–	4 (50.0%)
total leukocyte count	≥10*10^9/L	–	–	32 (39.0%)	–	2 (25.0%)
	<10*10^9/L	15 (18.3%)	35 (42.7%)	–	–	6 (75.0%)
FLT mutation	positive	1 (6.7%)	9 (25.7%)	16 (50.0%)	–	6 (75.0%)
	negative	14 (93.3%)	26 (74.3%)	16 (50.0%)	0.008	2 (25.0%)
hemorrhage	positive	5 (33.3%)	26 (74.3%)	20 (62.5%)	–	5 (62.5%)
	negative	10 (66.7%)	9 (25.7%)	12 (37.5%)	0.024	3 (37.5%)
fever	positive	7 (46.7%)	14 (40.0%)	16 (50.0%)	–	5 (62.5%)
	negative	8 (53.3%)	21 (60.0%)	16 (50.0%)	0.707	3 (37.5%)
fatigue	positive	9 (60.0%)	12 (34.3%)	13 (40.6%)	–	2 (25.0%)
	negative	6 (40.0%)	23 (65.7%)	19 (59.4%)	0.237	6 (75.0%)
splenomegaly	positive	3 (20.0%)	4 (11.4%)	7 (21.9%)	–	2 (25.0%)
	negative	12 (80.0%)	31 (88.6%)	25 (78.1%)	0.474	6 (75.0%)
lymph node enlargement	positive	0	2 (5.7%)	1 (3.1%)	–	1 (12.5%)
	negative	15 (100.0%)	33 (94.3%)	31 (96.9%)	1	7 (87.5%)
DS	positive	4 (26.7%)	6 (17.1%)	8 (25.0%)	–	1 (12.5%)
	negative	11 (73.3%)	29 (82.9%)	24 (75.0%)	0.644	7 (87.5%)

Date are presented as n (%) unless otherwise indicated.

DS, differentiation syndrome.

Twenty six patients had *FLT3* gene mutations (31.71%), which were 1/15 (6.67%), 9/35 (25.71%), and 16/32 (50.00%) in the low risk group, intermediate risk group and high-risk group respectively (*P*=0.008*)*. There was no statistically significant difference between the intermediate-risk group and the low-risk group (*P*=0.246), and there was a statistically significant difference between intermediate-risk group and the high-risk group (*P*=0.040), and there was a statistically significant difference between the high-risk group and the low-risk group (*P*=0.004).

There were 8 cases of children less than 12 years old (6 from intermediate-risk group, and 2 from high-risk group), male to female ratio 1:1, with main clinical features including fever and hemorrhage (*P*>0.05). One child patient experienced remission of DS during induction therapy. The *FLT3* mutation rates in adults (20/74,27%) and children (6/8,75%) were compared and the difference was found to be statistically significant(*P*=0.011).

### 3.2 Comparison of early patient outcomes for different treatment regimens

The early treatment regimens were divided into four groups, including all-trans retinoic acid (ATRA) group (group 1), ATRA combined with anthracycline group(group 2), ATRA combined with arsenic group (group 3), and ATRA combined with arsenic and anthracycline group (group 4). There were 15 patients in the low-risk group (3 received Group 1, 1 received Group 2, 6 received Group 3, and 5 received Group 4), 35 in the intermediate-risk group (0 received Group 1, 1 received Group 2, 14 received Group 3, and 20 received Group 4), and 32 in the high-risk group(3 received Group 1, 2 received Group 2, 5 received Group 3, and 22 received Group 4). The four groups were compared with each other, among which the mortality of the fourth group was at the bottom(4.26%) while the first and second groups reached the peak(50.00%). The fourth group had the highest CR(89.36%), followed closely by the third(76.00%) and then the second group(50.00%), of which three of the six patients in the first group died and three voluntarily dropped out of the induction treatment within a week for financial reasons. Group 4 was statistically different from group 1 and group 2 (*P=0.000,P=0.040*, respectively). Group 3 was statistically different from group 1 (*P=0.001).* Comparing the effects of using the ATRA combined with arsenic and anthracycline regimen in different risk groups with those of not using this regimen, we found that there was a statistical difference in the high-risk group (*P=0.001*) but no difference in the intermediate-risk group (*P=0.073*) (**Table 2**).

**Table 2:** Association between different regiments and early prognosis.

groups	number	death	CR	loss*
Group 1	6	3(50.00%)	0(0.00%)	3(50.00%)
Group 2	4	2(50.00%)	2(50.00%)	0(00.00%)
Group 3	25	5(20.00%)	19(76.00%)	1(4.00%)
Group 4	47	2(4.26%)	42(89.36%)	3(6.38%)
X^2^		26.717		
P		0.000		

Group 1= ATRA, Group 2=ATRA associated with anthracycline, Group 3=ATRA combined with arsenic, Group 4 =ATRA associated with arsenic and anthracycline. Loss*:Early abandonment or early loss of visits.

### 3.3 Induction stage and recurrence of patients

In this cohort of patients, 12 cases died during induction, with an early case mortality rate of 14.63%, and 7 patients stopped treatment or were lost to follow-up early. The causes of death included cerebral hemorrhage, sepsis complicated with multiple organ failure, pulmonary embolism, pulmonary embolism complicated with central nervous system (CNS) involvement, and pulmonary embolism complicated with pulmonary infection. Among them, cerebral hemorrhage accounted for a higher proportion of deaths (66.67%), with 8 cases dying of cerebral hemorrhage, 3 of them in the intermediate-risk group and 5 in the high-risk group. The early mortality rate of patients in the high-risk group was higher than that in other groups (*P*=0.041 and *P*=0.037) (**Table 3**).

**Table 3:** Association between different risk groups and early prognosis.

Risk group	Number	Loss*	Early deaths	CR
Low	15	4(26.67%)	0(0.00%)	11 (73.33%)
Intermediate	35	3(8.57%)	3 (8.57%)	29 (82.85%)
High	32	0(0.00%)	9 (28.13%)	23 (71.88%)
X^2^			7.386	1.367
P			0.022	0.483

CR, complete remission. Loss*:Early abandonment or early loss of visits.

The remaining 63 patients achieved complete remission after treatment, and the CR of the high-risk group was lower than that of the low-risk and intermediate-risk groups but did not reach statistical significance (*P*>0.05) (**Table 3**). Two patients experienced CNS relapse during the consolidation and maintenance phase of treatment after remission, of which one patient died and one patient achieved remission again after re-induction treatment. Among the 8 children less than 12 years old, 1 died of cerebral hemorrhage within 3 days of admission, 1 was lost to follow-up after 1 month of treatment, and the remaining 6 reached complete remission and were followed up for a long period of time until they were cured without recurrence.

### 3.4 Comparison of survival and disease-free survival in patients with different risk groups

**Figure 1:**
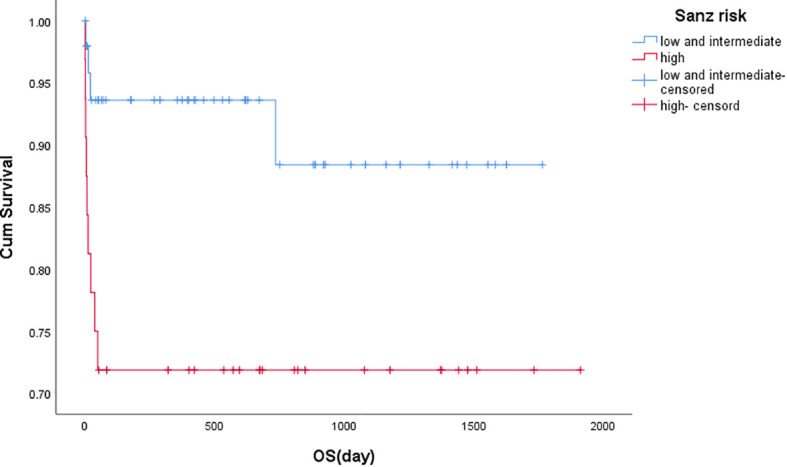
Kaplan-Meier estimates of overall survival based on risk groups. Kaplan-Meier curves showing 3 years landmark analysis for OS (Overall Survival) of patients with *PML/RARα* S fusion gene since the diagnosis of this disease. The low and intermediate risk group compared with the high risk group, and the difference was significant (*P*=0.019). Groups were compared by log-rank test.

3-year overall survival (OS) rate was higher in the low- and intermediate-risk group than that in the high-risk group (*P*=0.019), showing in **Figure 1** and **Table 4**.

**Table 4:** Association between different risk groups mid- and long-term prognosis.

Risk group	recurrence	3-year OS	3-year DFS
Low and Intermediate	2	86% ± 3.8%	88% ± 3.6%
High	0	70% ± 5.1%	69% ± 5.1%
P	–	0.019	0.017

**Figure 2:**
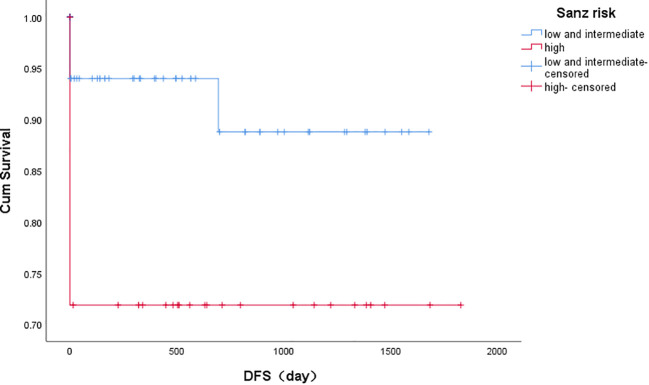
Kaplan-Meier estimates of disease-free survival based on risk groups. Note: Kaplan-Meier curves showing 3 years landmark analysis for DFS (disease-free survival) of patients with *PML/RARα* S fusion gene achieving the first remission. The low and intermediate risk group compared with the high risk group, and the difference was significant (*P*=0.017). Groups were compared by log-rank test.

3-year disease-free survival (DFS) was better in the low and intermediate-risk group than in the high-risk group (*P*=0.017), as shown in **Figure 2**.

## 4 Discussion

The treatment of APL has changed dramatically over the past decades. Anthracycline monotherapy was first introduced to treat APL in 1973 with some success, resulting in a 55-88% response rate in APL induction therapy, and in the mid-1980’s ATRA was used with great success in the treatment of APL, resulting in a 90% response rate in APL induction therapy. Subsequently, anthracycline- or arsenic-related therapies were introduced with ATRA and achieved good curative effect (4). Acute promyelocytic leukemia has special chromosomal translocations and fusion genes, and the fusion genes form three types, long (bcr1), variant (bcr2) and short (bcr3), depending on the *PML* breakpoints. Patients with *PML-RARα* short fusion gene have a high proportion of high-risk patients in risk stratification, more severe conditions, higher induced deaths and a poorer clinical prognosis (5). There are fewer clinical retrospective analyses in the literature that completely analyze bcr3 patients individually, so this study mainly analyzes the clinical features and induced therapeutic effect of bcr3 patients in the different risk groups as well as the reasons that cause outcomes.

In our study, the ratio of men to women was 1.05:1. At the same time, our present found that age and gender had no effect on this disease, similar to the previous study reported by Yedla (9), Dayama (10) and PETHEMA groups (11) investigating all APL.

Bleeding was the leading characteristic in our study followed by fever, which was different from the previous study reported by Yedla (9) and Bajpai (12) investigating all APL suggesting the most common presentation was fever. During induction therapy, there were 51 (62.20%) patients showing signs of bleeding, and 8 of them died of intracranial hemorrhage. Of the 8 patients, 3 were from the intermediate-risk group and 5 were from high-risk group. Therefore, for bcr3 patients, extra attention should be paid to the condition associated with bleeding as well as the changes of coagulation function and platelets during the early induction therapy stage, and once the coagulation function is abnormal and platelets are lowered, platelets should be transfused and coagulation factors or fibrinogen should be transfused in a timely manner (5).

In this study, the number of high-risk patients (39.02%) was almost equal to that of intermediate-risk patients (42.68%) and twice as many as low-risk patients (18.29%). This suggests that intermediate- and high-risk patients make up a major portion of short isoform patients and is one of the reasons for the relatively high mortality rate in those patients.

In this study, we noticed that the probability of DS was 21.95% which was slightly lower than the overall incidence of DS reported by Montesinos et al (13) (24.8%), Bajpai et al (12) (33%) and Yedla (9) (35.10%). This may suggest a lower incidence of DS in bcr3 patients but may also be due to the high early mortality rate in bcr3 patients (14). In this study, DS usually occurred within 2 weeks of taking ATRA or arsenic and ATRA. Once DS occurs, patients should stop ATRA/arsenic, and were treated with prompt diuresis, oxygen or BIPAP-assisted ventilation, and dexamethasone 10 mg IV twice a day for at least three days until the symptoms disappeared. All of the patients developing DS in this study had symptomatic relief, and none of them died of DS through timely treatment.

In the whole group the *FLT3* mutation frequency was 31.70%, and in the children group that is 75.0%. We found that the mutation rate of the low-risk group was 1.22% and intermediate-risk group was 10.98%, which were lower than that the high-risk group (19.51%), with a statistically significant difference. The mutation rate in bcr3 variants was slightly higher than 22.2% and 27% previously reported by Arrigoni P (15) and Kuty MA (16) respectively. Many studies considered the *FLT3* mutation rate had something to do with bcr3 isoform and high white blood cell count (17–19), which might explain the reason why *FLT3* mutation was higher than other studies. For the pediatric patients in this study, *FLT3* mutations were present in 75.00% with a mortality rate of 12.50% and no significant difference in male and female incidence rates (20), however, the number of children in our study was small. Current small molecule targeted agents against *FLT3* gene mutations bring new therapeutic approaches to patients with *FLT3* mutations (21).

82 patients were included in this study, 12 patients died during the induction therapy, 7 patients abandoned the treatment during the induction phase or missed the visit without complete remission, and all the patients who completed the early induction treatment were in remission, suggesting that those patients’ hazards mainly existed in the induction phase (22, 23) Intracranial hemorrhage (66.67%) was the leading cause of death during induction therapy followed by thrombotic events (25.00%) which is different to previous study focusing on all APL. Of the deaths, 3 patients from the intermediate-risk group and 9 from high-risk group suggested a higher risk of death among high risk APL patients with bcr3. The overall cure rate for APL is high, with current data showing as high as 90% (4), but a significant percentage of patients die within the first month of diagnosis, coinciding with our study. 63 patients were in complete remission in this study, with a complete remission rate of 76.83%. The complete remission rate of bcr3 patients is low compared to the remission rate of all APL patients, mainly because we did not find data on patients with bcr3 patients for comparison. The low complete remission rate is mainly due to characteristics of bcr3 patients themselves with more inclined to bleeding, higher risk stratification, and poorer clinical prognosis, and them died at an early stage and abandoned treatment or missed visits at early stage. Data from the Swedish Adult Acute Leukemia Registry showed an early mortality rate of 29%, mainly due to hemorrhage (10). The early mortality rate in our study was 14.63%, with cerebral hemorrhage as the main cause of death, which is a slightly lower mortality rate when compared to the literature data and may be due to the fact that we gave ATRA treatment in time at the time of the first suspicion of APL. Of the 63 patients who achieved complete remission, two had a central nervous system relapse in late follow-up, one of whom died due to cerebral herniation after central relapse (low-risk group), and the other reached complete remission again after follow-up treatment (intermediate-risk group). It indicated that those patients need to pay attention to consolidation and maintenance therapy and CNS prevention and treatment.

In addition, DFS and OS were higher in the low- and intermediate-risk group than in the high-risk group. However, the OS was lower compared with study reported by Sanz on account of more high- and intermediate-risk patients and more deaths from induction therapy as well as characteristics of bcr3 patients themselves.

In this study, the early treatment regimens were divided into four main groups. Six patients were treated with ATRA alone in group 1, of whom three died at an early stage. The responses of the patients to the different treatment regimens were compared, in which ATRA combined with arsenic and anthracycline had the lowest early mortality. The best regimen for APL patients with bcr-3 is still ATRA combined with arsenic and anthracycline agents (24–26). However, we compared the effects of using the ATRA combined with arsenic and anthracycline regimen in different risk groups with those of not using this regimen, finding that the high-risk group achieved better results with this regimen, so we recommend that high-risk bcr3 patients use this regimen for the relevant treatments.

In summary, this study found the probability of hemorrhage in APL patients with *PML/RARα* short fusion gene was higher and the clinical prognosis worse as the Sanz risk level increased. There were higher incidence of bleeding, cerebral hemorrhage mortality, *FLT3* gene mutation rate, early mortality rate and lower OS and DFS in the high-risk group. The main cause of death was cerebral hemorrhage, suggesting that bcr3 patients had a great risk of hemorrhage and a higher early mortality rate. In addition, for those patients, the clinical efficacy of ATRA combined with both arsenic and anthracycline drug was better.

## Data availability statement

The datasets presented in this study can be found in online repositories. The names of the repository/repositories and accession number(s) can be found in the article/supplementary material.

## Ethics statement

The studies involving humans were approved by ethics committee of Tongji Medical College, Huazhong University of Science and Technology. The studies were conducted in accordance with the local legislation and institutional requirements. Written informed consent for participation in this study was provided by the participants’ legal guardians/next of kin. Written informed consent was obtained from the individual(s), and minor(s)’ legal guardian/next of kin, for the publication of any potentially identifiable images or data included in this article.

## Author contributions

QH: Data curation, Writing – original draft. MZ: Conceptualization, Funding acquisition, Writing – review & editing. YZ: Funding acquisition, Supervision, Writing – review & editing.
